# Aerobic capacity evaluation of Sprague Dawley rats in treadmill running: comparison between protocols

**DOI:** 10.1590/1414-431X2025e13517

**Published:** 2025-04-14

**Authors:** C. Dellavechia-De-Carvalho, M.A. Rebelo, C. De-Moraes, M. Papoti

**Affiliations:** 1 Departamento de Ortopedia e Anestesiologia, Faculdade de Medicina de Ribeirão Preto, Universidade de São Paulo, Ribeirão Preto, SP, Brasil; 2 Departamento de Farmacologia, Faculdade de Ciências Medicinais, Universidade Estadual de Campinas, Campinas, SP, Brasil; 3 Escola de Educação Física e Esporte de Ribeirão Preto, Universidade de São Paulo, Ribeirão Preto, SP, Brasil

**Keywords:** Exercise, Animal, Performance, Test, Aerobic

## Abstract

The objective of the present study was to compare and test the applicability of different protocols for accessing aerobic capacity in Sprague Dawley rats using treadmill running. Fifteen 70-day-old adult Sprague Dawley rats (270-290 g) were used. After 5 days of adaptation to the treadmill, the animals underwent 7 days of evaluations with a 48-h interval between each protocol. On the first two days, they underwent, in random order, a graded exercise test, with (GXT2) or without (GXT1) blood sample collections to determine blood lactate concentrations and the anaerobic threshold. In the subsequent 4 days, they underwent continuous 30-min efforts to determine the maximal lactate steady state (MLSS) with the intensity prescribed in percentages of the maximum speed (MaxS) obtained in GXT1, and on the last day they underwent the minimum lactate (ML) protocol. The MaxS obtained in GXT2 was higher than in GXT1, and there was a moderate correlation (r=0.614, P=0.011) between them. In many cases, lactate and glucose blood concentrations did not show the expected kinetics, making aerobic capacity determination impossible using these protocols. MLSS showed a higher success rate compared to other protocols (MLSS=80%; GXT2=47%; ML=60%). In conclusion, with the MLSS protocol, it is only possible to measure time to exhaustion at each intensity, which does not exactly reflect aerobic capacity, and the use of blood lactate and glucose concentrations to evaluate the aerobic capacity of rats in incremental and ML treadmill running protocols is still discouraged.

## Introduction

The use of animal models is common in basic research and in exercise physiology studies when investigating the mechanisms induced by physical effort. The most commonly used animal models are rodents, rats, and mice, as they are small and easy to handle and the controlled conditions lead to low variation within the experimental groups and thus, enable the detection of small changes (1,2). The most commonly used physical exercise protocols are treadmill running and swimming, with the latter being the most used, both in studies that investigate the acute or chronic effects of physical exercise (3,4) and in protocols to determine effort intensity and exercise intensity in rats (1,5,6). The development of physical training protocols for animal models, both in swimming and running, is commonly based on parameters of aerobic capacity.

The most well-accepted protocol for aerobic capacity assessment, both in humans (7) and in animal models (8), is the maximal lactate steady state (MLSS), proposed by Beneke (9). The physiological basis of MLSS is comparable to the anaerobic threshold (AnT) theory (10), with MLSS characterized by the highest lactate blood concentration [La] that can be maintained in a steady state during submaximal, prolonged, and constant load exercise (10-12). The intensity corresponding to MLSS represents the highest submaximal exercise intensity that can be maintained in a steady state, in which blood lactate ([La]) remains stable during exercise (10,11). However, several days of assessment are required to determine this intensity, which can be considered a disadvantage of this protocol.

Another evaluation protocol also widely used to access aerobic capacity in animal models is the minimum lactate (ML) protocol proposed by Tegtbur et al. (13), which consists of three distinct phases. First, high-intensity efforts are carried out to induce hyperlactacidemia (phase 1), and then an 8-min recovery is required for the lactate to be transferred from the muscle to the bloodstream, so it can be quantified (phase 2). After recovery, an incremental test begins until exhaustion (phase 3). Blood lactate is expected to decrease during the first stages of the incremental test and to decrease in subsequent stages, giving the lactate kinetics curve a “U” shape. The intensity corresponding to the lowest point of this curve represents the ML intensity, which is an estimate of the equilibrium between lactate production and removal, similar to the MLSS (13,14). However, caution is necessary when applying an evaluation protocol that uses lactacidemia as a parameter, as it is known that manipulation of the variables of an incremental test in humans can alter both maximum speed (MaxS) (15) and the metabolic thresholds (16). The same effect could be expected in rodents, but this is not yet clear.

There are other protocols for determining aerobic capacity that, unlike MLSS and ML, require only one evaluation session. Among these, the graded exercise tests (GXT, in which there are progressive intensity increases) are widely used to determine the AnT (17). Typically, in these protocols, an exponential increase in lactate is observed with increasing effort intensity, and AnT is obtained from the inflection point of the [La]/intensity relationship (17,18). In addition to AnT, a GXT can provide another widely used parameter, the MaxS, which is the highest running intensity obtained in an incremental treadmill test and is commonly used to prescribe the intensity of running exercise sessions for animal models (19). In studies carried out in humans, in addition to [La], glycemia is also commonly measured for assessing aerobic capacity, since blood glucose has a “U“-shape kinetics both in incremental protocols (20) and ML protocols with an increase in intensity (21).

Although protocols to assess aerobic capacity are established, they are difficult to apply as they require lactate concentration quantification. In an attempt to offer a viable alternative for exercise intensity evaluation and monitoring in animal models, the objective of the present study was to compare and test the applicability of different protocols for assessing aerobic capacity in Sprague Dawley rats using treadmill running.

## Material and Methods

### Animals

To calculate the appropriate sample size, an *a priori* power analysis was conducted using G*Power (version 3.1.9.7, Germany). An effect size of 0.53 was used based on the previous results from Manchado-Gobatto et al. (22), which showed a significant effect on [La] at four distinct intensities. With a target power of 95% and an alpha level of P<0.05, 11 rats were required. However, considering a possible sample loss, which in previous studies was 25% on average, we decided to use fifteen seven-week-old adult Sprague Dawley rats (270-290 g), obtained from the Central Animal Facility of USP (Ribeirão Preto campus). Throughout the experiment, the animals were kept in collective cages, with four rats per cage and on a 12-h light/dark cycle. The experiments were carried out in the morning by the same researchers who maintained the animals in the cages. All animals received commercial food (Nuvilab, Brazil) and water *ad libitum*. The animals' food intake was controlled by weighing them daily before each procedure. All procedures in the present study were submitted and approved by the Ethics and Research Committee for the Use of Animals of the School of Physical Education and Sports of Ribeirão Preto (protocol No. 2016.1.462.90.7).

### Experimental design

**Figure 1:**
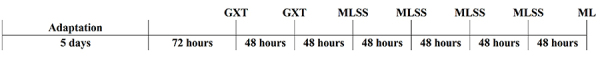
Chronological diagram of procedures. GXT: graded exercise test; MLSS: maximal lactate steady state; ML: minimum lactate test; MaxS: maximum speed.

All assessments were carried out on a treadmill (Gesam, Brazil), which has individual bays measuring 60×10×6.5 cm. The animals were adapted to the exercise for 5 days by running 10 to 60 min/day (Table 1). Seventy-two hours after adaptation, the animals were subjected to incremental evaluation protocols (GXT1 and GXT2) to determine AnT and MaxS, separated by 48 h. The order in which these protocols were carried out was randomized. Forty-eight hours after the second incremental protocol, the animals were subjected to 4 continuous efforts with constant loads separated by 48 h to determine the MLSS. Forty-eight hours after the last continuous effort, the animals were subjected to the ML test (Figure 1). For the GXT2, MLSS, and ML protocols, the success rate was calculated by dividing the number of tests that could be used to determine aerobic capacity by the number of tests in each protocol.

**Table 1:** Protocol for adapting animals to treadmill exercise. The graded exercise test was applied on day 5.

	Day 1	Day 2	Day 3	Day 4	Day 5
Velocity (m/min)	5	5	10	10	Start at 8 and progressively increase until exhaustion
Time (min)	30	60	30	60	10 to 25

### Collection and analysis of blood samples

Blood samples (25 μL) were collected by calibrated glass capillaries containing heparin from the distal end of the animals' tails and placed in microtubes containing 50 μL of sodium fluoride (1% NaF), homogenized, and kept at -4°C. [La] and glucose were determined using a Yellow Spring Instruments (YSI) Electrochemical Lactimeter, model 2300 Stat (Hong Kong).

### Graded exercise test 1 (GXT1) to determine the maximum running speed (MaxS)

Before the incremental test and exercise stimuli, the animals remained for 10 min in their respective treadmill stall for acclimatization. MaxS was determined during an incremental test with an initial speed of 8 m/min and increments of 3 m/min every 3 min until exhaustion. The exhaustion criterion used was the loss of the running pattern (i.e., interruption of the locomotion pattern, even with the application of stimuli). This same criterion was also adopted in the other evaluations. The maximum speed was determined from the equation:

$$MaxS=CS+\left(\frac{time}{180}\right)\times I$$ (Eq. 1)

where CS is the speed of the last completed stage, I is the velocity increments between stages, time is the time in the stage in which exhaustion occurred in seconds, and the constant 180 refers to the duration (in seconds) of the test stages (23).

### Graded exercise test 2 (GXT2) to determine the anaerobic threshold (AnT)

To determine AnT, we used the same protocol used in GXT1, but with blood collections at rest and at the end of each stage. AnT was determined using the bi-segmented method (24-26) by two experienced evaluators. In case of discrepancy, a third evaluator made the determination. To determine the glycemic threshold, a second-order polynomial adjustment was applied to the relationship between glucose and GXT intensity, and the glucose minimum was considered as the minimum point or zero derivative of the curve formed.

### Maximal lactate steady state (MLSS)

To determine the MLSS, the animals were subjected to 4 continuous efforts of 30 min each with intervals of 48 h between efforts at intensities corresponding to 60, 70, 80, and 90% of the MaxS obtained at GXT1, since the starting point for the first evaluation session is closer to real MLSS intensity than the determination of the intensities. With this strategy, fewer sessions are required. We based the choice of these intensities on the study by Ferreira et al. (19), which showed that MLSS intensity corresponded to 60% of MaxS, obtained in a protocol similar to GXT1. The order in which these efforts were carried out was randomized. Blood collections were performed every 10 min. The intensity corresponding to MLSS was considered the highest effort intensity at which the difference in [La] between the 30th and 10th was less than 1 mM (5).

### Minimum lactate (ML) test

We used the hyperlactacidemia induction phase of the ML test following the protocol used by de Araújo et al. (1), which consists of two efforts, the first lasting 30 s and the second after 30 s of passive recovery until exhaustion. The intensity of these efforts was fixed at 20 m/min. This speed represents 121, 106, and 108% of the MaxS of these animals in the GXT1, GXT2, and ML protocols, respectively. After the induction phase, the animals remained in passive recovery for 8 min, and blood samples were collected in the 3rd, 5th, and 7th min of recovery. Immediately after 8 min, the animals were subjected to an incremental protocol similar to GXT2. From both the [La] and glucose values, a second-order polynomial adjustment was applied to the relationship of these variables and the intensity of the GXT and the ML point was considered to be the minimum point or zero derivative of the formed curve.

### Statistical analysis

Data are reported as means±SD. Data normality was measured and confirmed using the Shapiro-Wilk test. To compare the methods, one-way repeated measures ANOVA and the Pearson correlation coefficient (r) test were used to assess possible correlations. The significance level was set at P<0.05.

## Results

**Figure 2:**
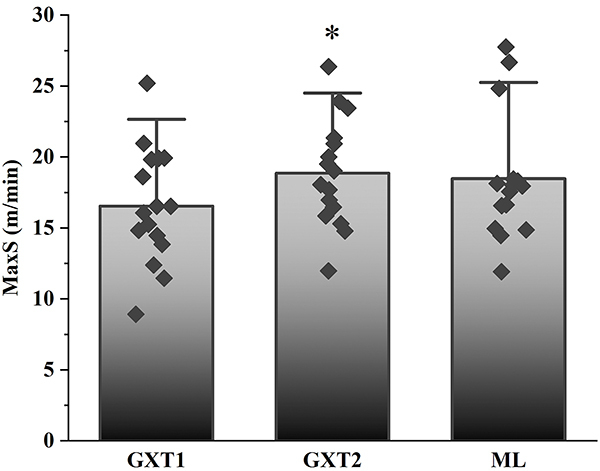
Maximum speed (MaxS) reached in incremental protocols. Data are reported as means and SD. *P<0.05 compared to GST1; ANOVA. GXT1: incremental test without pauses for blood collections; GXT2: incremental test with pauses for blood collections; ML: minimum lactate test.

The MaxS achieved in the incremental tests was 16.5±4.1, 18.9±3.8, and 18.5±4.5 m/min, and the time to exhaustion was 11.2±3.9, 13.8±3.7, and 13.5±4.4 min in the GXT1, GXT2, and ML protocols, respectively. The MaxS in GXT2 was higher than in GXT1 (P<0.05) (Figure 2). Furthermore, a moderate positive correlation was found between GXT1 and GXT2 (P<0.05) (Table 2).

**Table 2:** Pearson correlation between the maximum speeds at each incremental protocol.

	R	P
GXT1 and GXT2	0.614	0.011*
GXT1 and ML	0.379	0.164
GXT2 and ML	0.390	0.151

R: correlation coefficient; GXT1: incremental test without pause for blood collection; GXT2: incremental test with pause for blood collection; ML: minimum lactate test. *P<0.05.

**Figure 3:**
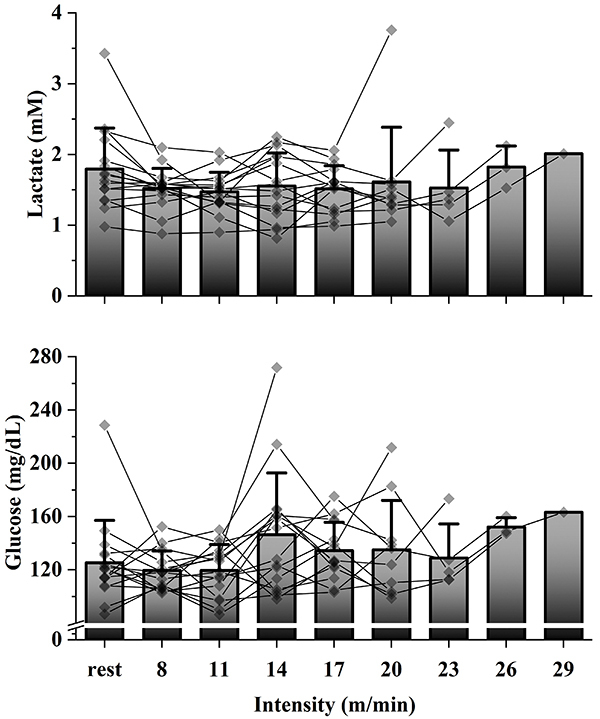
Mean and individual lactate and glucose values from the graded exercise test 2. Each line represents an individual animal.

**Figure 4:**
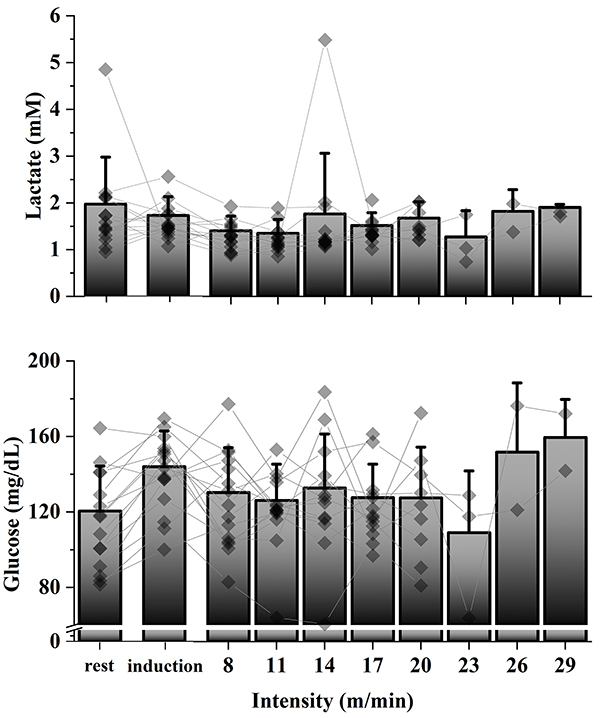
Average and individual lactate and glucose values from the minimum lactate test. Each line represents an individual animal.

Figures 3 and 4 show the individual values and average at each point of lactate and glucose in the GXT2 and ML protocols, respectively. By a visual inspection of these two variables, we obtained a low success rate in accessing the aerobic capacity in these protocols (Table 3).

**Table 3:** Intensity corresponding to aerobic capacity during the three protocols tested.

	MLSS	GXT2	ML
Velocity (m/min)	12.3±3.2	19.4±4.8	11.2±1.3
%Vmax	74.2±5.1%	91.4±7.7%	66.8±9.7%
N	12	7	9
Success (%)	80	47	60

Data are reported as means and SD. MLSS: maximal lactate steady state; GXT2: graded exercise test 2; ML: minimum lactate test; Success: percentage of success in determining aerobic capacity.

## Discussion

The methods used in the present study sought to define the relationships between aerobic capacity assessment protocols and the use of GXT without blood collection to determine lactacidemia as a reliable method for evaluating and controlling the intensity through these protocols in an animal model based on its relationship with gold standard tests (i.e., MLSS). Our results point to a large variation between the results of the different protocols, mainly due to the low success rate in determining the aerobic capacity and the fact that [La] did not present the expected behavior.

Variables that represent aerobic capacity and power from assessment protocols are extremely important for individual prescription and quantification of training loads. The most accurate method for assessing the aerobic capacity is MLSS, so this protocol can be used for this purpose as well as for other experimental protocol standardization. However, this method has a limiting factor, which is the relatively high number of assessments required, which makes its use unfeasible for prescription, monitoring, and measuring protocols. In the present study, a satisfactory success rate (80%) was observed for the MLSS protocol based on the pre-established criteria, showing that this protocol could be used for assessing aerobic capacity in rats. Based on the [La] found in the other protocols, it is not certain that increases in intensity reflect [La] increases as well, although other studies point to the effectiveness of this protocol in an animal model in running exercise (22,27) and in swimming (6).

The most commonly used alternative protocols to assess aerobic capacity (e.g., incremental protocol and minimum lactate) require moments of high-intensity effort and blood collections to measure lactate and glucose. Behavioral studies indicate that these conditions can increase stress levels and, consequently, lead to inflammatory conditions in animals such as serum high-sensitive C-reactive protein, tumor necrosis factor α, and adiponectin (28). However, these protocols have the main advantage of requiring only one session, as well as the possibility of identifying a maximum effort parameter in the same test. We tested two incremental protocols and the ML protocol to determine the AnT, and from these tests we also obtained MaxS, a parameter commonly used to prescribe exercise in animal models. We used a protocol similar to that used by Ferreira et al. (19) in mice at GXT1, but we decided to use the initial speed of 8 m/min, following a recent standardized evaluation protocol for rats (29).

We did not observe any difference in the MaxS obtained in the ML protocol compared to the incremental protocols. However, the MaxS was lower for GXT1 than for GXT2. It is logical to consider that this difference was due to the fact that at GXT2 there were small breaks (considered recovery breaks) for blood collection, which may have contributed to the better performance. Even so, the values obtained in GXT1 and GXT2 showed a moderate correlation. Ferreira et al. (19) showed that MLSS in mice is on average 60% of MaxS, while in the present study, the intensity corresponding to MLSS was 74% of MaxS. Due to the low success rate in assessing aerobic capacity in GXT1 and ML, the percentage of their corresponding intensities may not represent the reality for this group of animals.

We sought to identify the intensities corresponding to AnT using the GXT2 and ML tests, in which an exponential increase in lactate and a U-shaped kinetics, respectively, were expected, as occurs in humans (30-32) or even in rats in swimming (33). However, blood lactate did not show the expected kinetics in these protocols, making it impossible to analyze and identify the intensity corresponding to AnT in many cases of the present study using these methods. Langfort et al. (34) and Soya et al. (35) successfully identified the expected kinetics of lactate increase in an incremental running protocol in Wistar rats. However, Carvalho et al. (36) found [La] kinetics similar to that of the present study in female Wistar rats during treadmill running. Some studies were successful in assessing aerobic capacity using the minimum lactate test in swimming in rats (1,37) and mice (5), but there are no studies that had the same success when running. Therefore, caution is needed when choosing the type of physical exercise in which these protocols will be applied, since lactate may not present the expected kinetics during running. We can speculate that running exercise may cause some additional physiological stress to the animals, so that lactate analysis is impaired. A possible reason for this could be the exhaustion caused by the exercise-induced changes in the core body temperature of rats (38,39).

In the present study, blood glucose, as lactate, also did not show the expected kinetics, making aerobic capacity assessment impossible based on this variable. There are no studies that measured blood glucose in rodents for this purpose. Therefore, based on the results of the present study, the use of glucose for the assessment of aerobic capacity is discouraged.

Some possible limitations regarding the present study are: i) the GXT protocols vary widely in the literature, which limits the comparison amongst studies; ii) the fact there were no breaks between stages in GXT1 made its comparison with GXT2 difficult. Considering the difficulties found in the present study, future studies are needed to find practical and reliable protocols for aerobic capacity assessment in running models in rats.

## Conclusion

In conclusion, with the MLSS protocol, it is only possible to measure time to exhaustion at each intensity, which does not exactly reflect aerobic capacity. The use of [La] and glucose for aerobic capacity assessment in incremental and ML protocols is still discouraged, but the MaxS obtained in these protocols is an interesting parameter that can be used in evaluation protocols and subsequent prescriptions for running intensity in rats.
